# Function of Uric Acid Transporters and Their Inhibitors in Hyperuricaemia

**DOI:** 10.3389/fphar.2021.667753

**Published:** 2021-07-14

**Authors:** Hao-lu Sun, Yi-wan Wu, He-ge Bian, Hui Yang, Heng Wang, Xiao-ming Meng, Juan Jin

**Affiliations:** ^1^ Department of Pharmacology, Anhui Medical University, Hefei, China; ^2^ Inflammation and Immune Mediated Diseases Laboratory of Anhui Province, Anhui Institute of Innovative Drugs, School of Pharmacy, Anhui Medical University, Hefei, China

**Keywords:** uric acid, transporters, gene, hyperuricaemia, inhibitor

## Abstract

Disorders of uric acid metabolism may be associated with pathological processes in many diseases, including diabetes mellitus, cardiovascular disease, and kidney disease. These diseases can further promote uric acid accumulation in the body, leading to a vicious cycle. Preliminary studies have proven many mechanisms such as oxidative stress, lipid metabolism disorders, and rennin angiotensin axis involving in the progression of hyperuricaemia-related diseases. However, there is still lack of effective clinical treatment for hyperuricaemia. According to previous research results, NPT1, NPT4, OAT1, OAT2, OAT3, OAT4, URAT1, GLUT9, ABCG2, PDZK1, these urate transports are closely related to serum uric acid level. Targeting at urate transporters and urate-lowering drugs can enhance our understanding of hyperuricaemia and hyperuricaemia-related diseases. This review may put forward essential references or cross references to be contributed to further elucidate traditional and novel urate-lowering drugs benefits as well as provides theoretical support for the scientific research on hyperuricemia and related diseases.

## Introduction

With lifestyle changes, hyperuricaemia has become common around the world. 85–90% of hyperuricaemia patients have no clinical features. This stage is asymptomatic hyperuricaemia. Over time, long-term high serum uric acid (SUA) may cause many complications. Current studies have shown that 1) anomalous high SUA level is likely to induce a series of cardiovascular diseases, and acts as an independent risk factor for cardiovascular diseases, including atherosclerosis, hypertension, and coronary heart disease (Edwards, 2009); 2) obesity can cause hyperuricaemia, further leading to lipid metabolism disorder and chronic diseases (Fernandes Silva et al., 2019; Jingzhe Han et al., 2019); 3) in patients with diabetes, high uric acid (UA) further damages pancreatic cells and worsens diabetic condition (Changgui Li and Chang, 2013; Yun-Hong Lu et al., 2020). In summary, hyperuricaemia has become a key risk factor for development of many serious diseases.

Hyperuricaemia occurs due to alterations in urate production or excretion. UA is the final metabolite of purines and mainly excreted in the body by the kidney and intestine. The kidney excretes about two-thirds while the gastrointestinal tract excretes one-third of the UA load (Jessica Maiuolo et al., 2016). Most UA is filtered from glomerular, while renal tubules reabsorption and secretion regulate the amount of urate excretion (Jessica Maiuolo et al., 2016). The proximal tubule is the site of UA reabsorption and excretion. About 90% UA is reabsorbed into blood (Jessica Maiuolo et al., 2016). Urate transporters are mostly located in the proximal tubules of the kidney and play key roles in reabsorption and excretion of UA. In this review, we discuss the molecular mechanisms of urate transports and their inhibitors on hyperuricaemia-associated diseases. This study not only shows that urate transports play considerable roles in the progression of hyperuricaemia-associated diseases but also indicates that they may be used as therapeutic targets.

## Uric Acid

**FIGURE 1:**
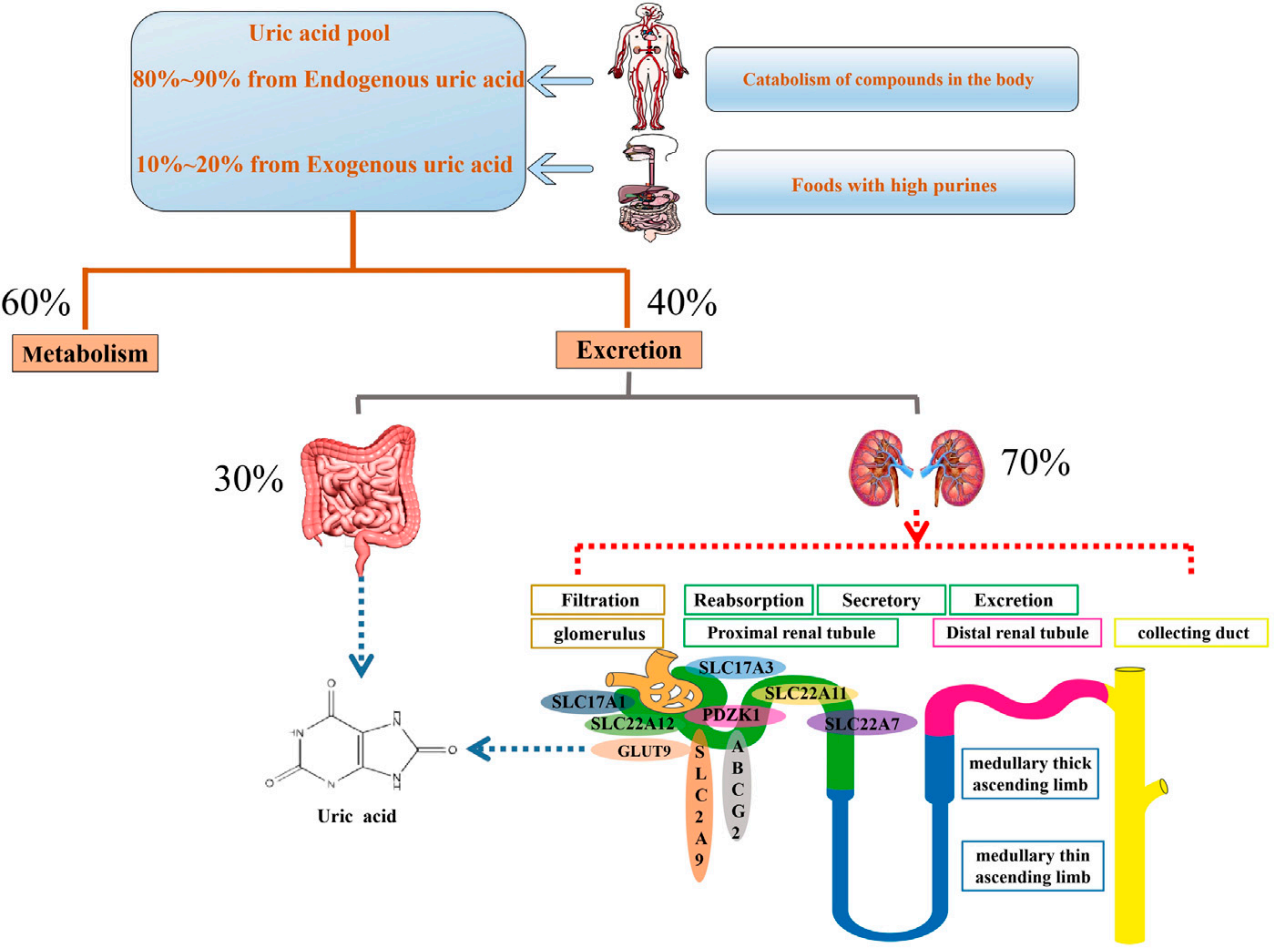
Mainly physiological progression of uric acid in the body. Serum uric acid is original from uptake of foods containing a high level of purines as well as catabolism of proteins and other compounds in the human body. About 60% uric acid involves in metabolism process and the rest of uric acid is excreted through the gut and urethra. Urethral excretion is the main way. A series of urate transporters including SLC and ABC transporters expressed in urethra, especially the proximal convoluted tubules, maintain urate homeostasis.

At present, two sources of UA are recognized: 1) uptake of foods containing a high level of purines; 2) catabolism of proteins and other compounds in the human body. In long-term evolution, human UA kinase factors and a series of promoter gene mutations have caused humans to have higher UA levels than other mammals (Figure 1) (Edwards, 2009). Physiologically, it involves in many enzymes and hormones, such as xanthine oxidase, reproductive hormone, growth hormone, thyroid hormone, etc (Chengfu Xu et al., 2015; Liangshan Mu et al., 2018; Linqiang Ma et al., 2018; Shuang Liang et al., 2018). UA, as scavenger of oxygen radical, contributes to approximate 60% of plasma antioxidant activity and maintains the stability of blood pressure and antioxidant stress (Nieto et al., 2000; Wang et al., 2020). In addition, it prevents the oxidation of low-density lipoproteins and the inactivation of superoxide dismutase (Rudan et al., 2010). This antioxidant activity displays the protective roles of UA action under physiological environment. However, the abnormal UA level may be is correlative with many diseases.

## Hyperuricaemia

Hyperuricaemia is due to the broken balance between production and complex processes of secretion and reabsorption of UA (So and Thorens, 2010). A growing number of publications demonstrate that hyperuricaemia has been proved to be a risk for multiple diseases including gout, chronic kidney disease (CKD), cardiovascular diseases. Despite the importance of hyperuricaemia, the definition of hyperuricaemia remains inconclusive. Generally, abnormal SUA is higher in men than in women (Bardin and Richette, 2014). Hyperuricaemia is defined increased SUA as above 7 mg/dl in men and above 6 mg/dl in women in many studies (Zhu et al., 2011; Bardin and Richette, 2014)). High intake of purine-rich foods (such as alcohol) or those that can lead to increased purine levels (such as fructose) can also contribute to hyperuricaemia (Xiao et al., 2018; Go et al., 2019; Hoogerland et al., 2020). Genetic variations induced by multiple factors are also the key cause of hyperuricaemia (Guo et al., 2005; Mount and Mandal, 2019). Moreover, SUA concentration is negatively correlated with maximal oxygen uptake and positive correlation with carbon dioxide in patients with chronic heart failure (Leyva et al., 1997). Hypoxia leads to the accumulation of UA precursors and the activation of xanthine oxidase/dehydrogenase further increase the level of UA in the body (Hassoun et al., 1992). During hypoxia, glycolysis accelerates the production of UA in patients with heart failure (Swan et al., 1994). NO, an endothelial cell-derived relaxing factor produced by endothelial cells is another important factor affecting uric acid. Decreased levels of NO are leading to increased UA and insulin resistance occurence (Cook et al., 2004; Gehr, 2004; So and Thorens, 2010). Chronic elevation of SUA level leads to the formation and deposition of monosodium urate (MSU) crystals leads to inflammatory reaction and tissue injury in many organs such as joint, kidney, and heart (Bardin and Richette, 2014). Together, the available information indicates that the regulation of UA level is complex and may explain the association with hyperuricaemia, gout, the metabolic syndrome, cardiovascular disease, and renal disease.

## Hyperuricaemia-Related Diseases

### Gout

Strong epidemiological evidence demonstrates that the prevalence of gout is growing worldwide. Gout is a chronic disease caused by MSU crystal deposition, while hyperuricaemia is the major risk factor (Dalbeth et al., 2019). Over time, prolonged hyperuricaemia may result in more frequent and severe symptoms of gout (Dalbeth et al., 2019). In patients with established gout, elevated SUA is associated with increased risk of recurrent gout (Shiozawa et al., 2017). Despite advances in understanding the pathophysiology of gout, it remains common and challenging which may be associated with untimely treatment and lack of understanding about the role of urate-lowering therapy (Stamp and Dalbeth, 2019). For the treatment of gout, allopurinol remains the first-line urate-lowering therapy, with febuxostat regarded as an proper alternative in clinical practice (Stamp and Dalbeth, 2019). Lifestyle modification including reduced intake of purine-rich foods, weight loss, avoidance of fructose and alcohol has also been considered an vital aspect of gout management (Jeyaruban et al., 2016). Using available data, keeping the balance between excretion and overproduction of SUA is reasonable for prevention and management of gout.

### Chronic Kidney Disease (CKD)

Increasing evidence indicates that SUA is enhanced in patients with CKD (Liu et al., 2015). Epidemiological studies have reported the association between hyperuriceamia and CKD (Alobaidi et al., 2021). About 20–60% of patients with established gout have renal dysfunction (Kang et al., 2002). It is a risk marker and contributes to the development of glomerulosclerosis and interstitial fibrosis (Kang et al., 2002; Liu et al., 2015). Hyperuriceamia may aggravate kidney damage through RAS activation which is an important mediator of kidney disease progression or through developing hypertension by increasing salt sensitivity (Kang et al., 2002; Watanabe et al., 2002). Furthermore, it induces macrophage infiltration, renal tubular epithelial to mesenchymal transition, as well as an increased expression of inflammatory mediators (Balakumar et al., 2020). For the management of CKD, many investigations indicate that urate-lowering therapy slows and delays the development of CKD (Sonoda et al., 2011; Shi et al., 2012; Liu et al., 2015). In addition, these findings are contributed to screen and manage individuals with an elevated risk of CKD development.

### Cardiovascular Diseases

Hyperuricaemia has been considered as not only a risk factor for human cardiovascular diseases such as myocardial infarction, hypertension, but also a co-variable of other known risk factors for cardiac deaths and coronary heart disease (Culleton et al., 1999; So and Thorens, 2010; Tian et al., 2021). In addition, hyperuriceamia is also demonstrated as an independent risk factor for cardiovascular mortality (Krishnan et al., 2008). It is reported that the risk of myocardial infarction enhances with higher cumulative UA. Early cumulative UA contributed more to myocardial infarction risk than later cumulative UA with the same overall cumulative exposure (Tian et al., 2021). UA further contributes to the development of hypertension in obesity (DeMarco et al., 2014). In a cross-sectional study of the association between hyperuricaemia, hypertension and ischemic stroke, higher risk is found even after full adjustment in participants with hyperuricaemia and hypertension (Sun et al., 2021). These findings highlight the importance of optimal SUA control in preventing cardiovascular diseases.

### Metabolic Syndrome

High UA level is found in patients with metabolic syndrome (Keskin et al., 2021). Insulin resistance can elevate UA by reducing renal urate clearance (Facchini et al., 1991). Increasing studies verify that insulin resistance is usually accompanied by an increased UA and high UA could induce insulin resistance (Bassanese et al., 2021; Jiao et al., 2021). While insulin resistance leads to a significant increase in the expression of urate transport-related proteins, an increase in urate reabsorption and an increase in SUA levels (Zhang et al., 2021). Therefore, hyperuricaemia and insulin resistance may promote each other. Reducing the expression of UA transporter proteins in the setting of insulin resistance down regulates blood UA levels (Zhang et al., 2021).

When cells are induced to differentiate into adipocytes, the physiological concentration of uric acid is further increased (So and Thorens, 2010). Hyperuricaemia in obese people is mainly caused by impaired renal uric acid clearance, not overproduction (Enomoto et al., 2002). This may be related to the mechanism of reactive oxygen species (ROS) production involving in NADPH oxidase activation (So and Thorens, 2010). Excretion of UA is inversely associated with leptin as a predictive marker for metabolic syndrome secreted by adipose tissue (Boden et al., 1997; Saad et al., 1998; D’Elia et al., 2020; Ghadge and Khaire, 2019). UA may also regulate leptin levels by changing leptin gene expression or decreasing leptin clearance (Fruehwald-Schultes et al., 1999). In conclusion, these findings suggest that some substances like leptin may involve in the crosstalk between metabolic syndrome and hyperuricaemia.

### Neurodegenerative Diseases

Hyperuriceamia displays an important causative effect in multiple diseases including gout, CKD, cardiovascular diseases. In contrast, it shows a protective role in neurodegenerative disorders such as Alzheimer’s disease (Tana et al., 2018; Mandal and Mount, 2019). Previous studies indicate that reduced SUA levels in the body can lead to an increased risk of Alzheimer’s disease, Parkinson’s disease, multiple sclerosis, schizophrenia, and dementia (Ascherio et al., 2009; Du et al., 2016; Ye et al., 2016). These findings may be associated with the antioxidant effects that UA may display in neurodegenerative diseases (Tana et al., 2018). Controversially, there is conflicting evidence about SUA in cognitive decline in patients with vascular or mixed dementia (Tana et al., 2018). Hong et al. find a lower risk of developing vascular dementia in patients with established gout (Hong et al., 2015). However, Khan et al. did not find a significant association between them (Khan et al., 2016). One recent study indicates a significant risk of vascular or mixed dementia in patients with higher SUA (Latourte et al., 2018). Thus, UA may represent to be a complex mediator of influencing cognitive function in different dementia types. The specific contribution of UA on neurodegenerative disorders needs to be further clarified.

The strong association between SUA and cognition could be indicated also by the relationship between certain genetic abnormalities of the urate transporters and nerve injury. *SLC22A12* gene encoding for the urate transporter hURAT1 defects leads to primary renal hypouricemia characterized by increased UA excretion from a reduced reabsorption (Tana et al., 2018). Variations in *SLC2A9* gene, encoding the urate transporter GLUT9, are closely related to human cognition and neurodegenerative diseases (Houlihan et al., 2010; Mandal and Mount, 2019). ITM2B, a GLUT9-interacting protein, inhibited urate influx and stimulated urate efflux (Mandal and Mount, 2019). These data demonstrate that there may be some regulators as potential molecular links between UA homeostasis and neurodegenerative disorders.

## Urate Transporters and Genetics of Urate Transporter Pathologies

**FIGURE 2:**
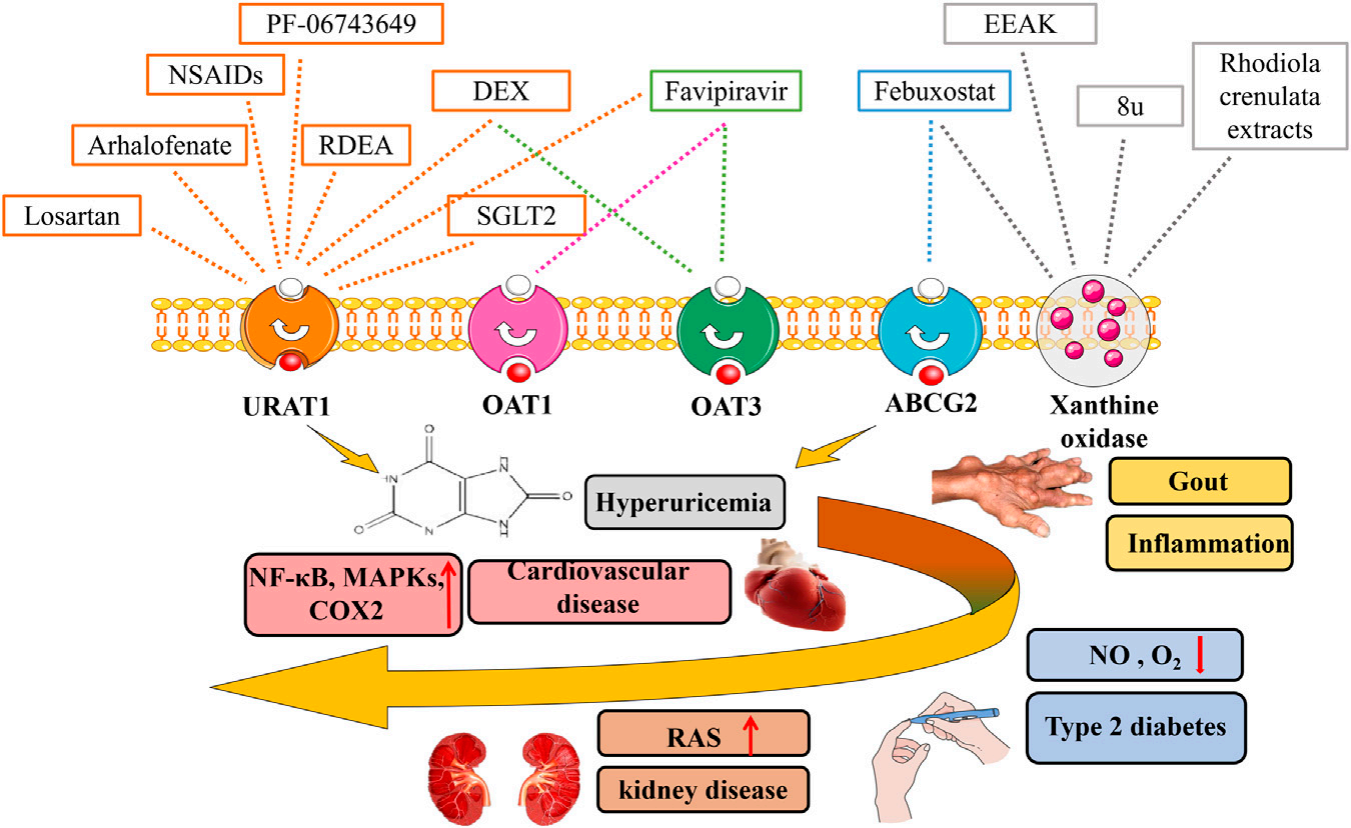
Urat-lowering drugs in hyperuricaemia-related diseases. Hyperuricaemia has been proven to be associated with multiple diseases including gout, chronic kidney disease, cardiovascular diseases, metabolic syndrome. The discovery of urate transporters provides new ideas for the development of drugs for the research of hyperuricaemia. In addition to urate transport inhibitors, xanthine oxidase inhibitors, SGLT2 inhibitors, as well as novel urat-lowering drugs like EEAK, Favipiravir, PF-06743649 have been summarized.

A series of urate transporters including SLC and ABC transporters as well as several multispecific drug transporters (e.g., OAT1, OAT2, and ABCG2) maintain UA homeostasis (Figure 2) (Tomita et al., 2000; Edwards, 2009; Nigam and Bhatnagar, 2018). Over time, it has become apparent that altered urate transport, both in the gut and the kidneys, has a vital role in the pathogenesis of hyperuricaemia-associated diseases. Thus, the optimization of UA level can be regarded as a systemic issue. Recent studies have suggested that urate transporters mutations in genes and related sites directly affect urate reabsorption and excretion (Guo et al., 2005; Puig and Martínez, 2008). Compared with the modern environment, genetic factors on urate transporters have a larger effect on variation in serum urate concentrations (Major et al., 2018). Exploration of these transporters and related genes sites are important to regulate and achieve target serum urate (Table 1).

**TABLE 1:** Characteristics of Urate transporters.

Transporter	Location in kidney	Mutation	Function of transporters
***SLC2A9* (GLUT9)**	Apical and basolateral membranes of the renal proximal tubule	*R198W, R380C, P412R* Anzai et al. (2008); Dinour et al. (2010); Kawamura et al. (2011)	Urate reabsorption
***SLC16A9* (MCT9)**	In the kidney and adrenal gland	*rs1171614* Butler et al. (2021)	Related to UA levels
		*rs2242206* Nakayama et al. (2013)	
***SLC17A1* (NPT1)**	Apical membrane of the renal proximal tubule	*rs1165196* Chiba et al. (2015); Sakiyama et al. (2016)	Urate absorption and efflux
		*I269T* Sakiyama et al. (2016)	
***SLC17A3* (NPT4)**	Apical side of renal tubules	*rs9393672* Sakiyama et al. (2016); Jutabha et al. (2010); Riches et al. (2009)	Urate excretion
		*rs942379* Sakiyama et al. (2016); Jutabha et al. (2010); Riches et al. (2009)	
***SLC22A6* (OAT1)**	Basolateral side of the proximal tubule	----	Urate excretion
***SLC22A7* (OAT2)**	Mainly distributed in the kidney	*C329T, G571A, G1520A* Xu et al. (2005); Kato et al. (2015)	Urate secretion
***SLC22A8* (OAT3)**	In the basolateral side of the proximal tubule	----	Urate excretion
***SLC22A11* (OAT4)**	Proximal tubules of the kidney and other marginal epithelia	*rs2078267* Kenny et al. (2011); Flynn et al. (2013)	Urate reabsorption
		*rs2186571* Kenny et al. (2011); Flynn et al. (2013)	
		*rs17299124* Sakiyama et al. (2014)	
		*rs17300741* Sakiyama et al. (2014)	
**SLC22A12 (URAT1)**	Apical membrane of the proximal tubule	*rs475688* Pavelcova et al. (2021)	Urate reabsorption
**ABCG2**	Renal tubules and mesentery	*V12M, Q126X, Q141K, G268R, S441N, and F506SfsX4* Matsuo et al. (2009)	Urate excretion
**PDZK1**	Apical membrane of the kidney proximal tubule	*rs12129861* Butler et al. (2021)	Regulate the transport and activity of various transport proteins in the proximal tubules

### *SLC* Transporters

#### GLUT9 (*SLC2A9*)

GLUT9 encoded by *SLC2A9* gene is widely present in the proximal tubule in human kidney (Kimura et al., 2014). It has two splice variant isoforms, GLUT9a (540 amino acids encoded by 12 exons) and GLUT9b (512 amino acids encoded by 13 exons). In human, GLUT9a is expressed in the basolateral membrane of proximal tubules of human kidney, whereas, GLUT9b is expressed at the apical membrane of the collecting duct (So and Thorens, 2010). GLUT9 initially identified as the glucose transporter serves acritical role in urate reabsorption (Ebert et al., 2017). Genetic inactivation in mice induces moderate hyperuricaemia and massive renal excretion of urate. Accumulating studies report that hypouriceamia has been associated with mutations in the *SLC2A9* gene. For example, L75R mutation and 36-kb deletion present in two different families of hypouriceamic patients; R198W, R380C, as well as P412R mutation are found in hypouriceamic patients, respectively (Anzai et al., 2008; Dinour et al., 2010; Kawamura et al., 2011).

#### MCT9 (*SLC16A9*)

The human *SLC16* protein, also known as human monocarpboxylic acid transporters (hMCTs), contains 14 members and mediates the transport of monocarboxylates through the plasma membrane (Futagi et al., 2020). MCTs are generally divided into two categories, the H^+^-sensitive and H^+^-non-sensitive transporters. MCT9 belongs to the latter and is ubiquitous with the highest expression level in the kidney and adrenal gland (Harst et al., 2010; Roshanbin et al., 2016; Console et al., 2020). *rs1171614* and *rs2242206* variants has been reported to be associated with SUA levels and the risk of kidney overload gout (Nakayama et al., 2013; Butler et al., 2021). At present, many members of the *SLC16* family have never been studied. Although MCT9 has been shown to be related to UA levels, its mechanism of action and location are still unclear. Further genetic and functional study of MCT9 is necessary.

#### NPT1 (*SLC17A1*)

The *SLC17A1*, a membrane protein, is the first member of the SLC17 phosphate transporter family (Zhang et al., 2018). *SCL17A1* gene encoding NPT1 transports various substrates including UA (Chiba et al., 2015). A previous analysis shows that NPT1 mainly expressed in the kidney is localised to the apical membrane of the renal proximal tubule (JadeHollis-Moffatt et al., 2012; Chiba et al., 2015).

##### Function of NPT1 (*SLC17A1*)

NPT1, weakly to moderately correlate with altered UA levels, mediates the absorption of UA when the plasma membrane is depolarised by high concentration of exogenous potassium (Bhatnagar et al., 2016). At the same time, NPT1 is conducive to the efflux of UA. When the cell membrane presents a negative potential phase, NPT1 mediates UA efflux (Chiba et al., 2015). NPT1 as a Cl^−^-dependent urate transport, has two transportation activities, namely the of Na^+^/phosphate co-transport; moreover, anion conductance is important to discriminate the mechanistic differences between the two activities and Δψ-driven anion transport activity (Wright and Dantzler, 2004; Iharada et al., 2010).

##### Mutation Loci of the NPT1 (*SLC17A1*) Gene

The study selects 545 Japanese men with gout as a model group and 1,115 healthy men as a normal control group to investigate mutations in the NPT1 *rs1165196* and *I269T* genes (Chiba et al., 2015). Functional analysis shows that NPT1 *rs1165196* variants significantly reduce the risk of renal under excretion gout and enhance the renal urate secretion (Chiba et al., 2015; Sakiyama et al., 2016). Interestingly, *rs1165196* variants have little effect onpatients with normal renal excretion (JadeHollis-Moffatt et al., 2012; Chiba et al., 2015; Sakiyama et al., 2016). *I269T* (a common missense variant of NPT1) mutations increase the maximum volume by increasing the turnover rate of the urate transport and output tomitigate gouty risk, but do not change NPT1 membrane expressions (Sakiyama et al., 2016). Compared with NPT1 wild type, *I269T* might have faster conformation changes leading to enhance renal urate export (Sakiyama et al., 2016).

#### NPT4 (*SLC17A3*)

Human sodium phosphate co-transporter type 4 (NPT4/SLC17A3) is a multi-specific organic anion efflux transporter expressed in the kidneys and liver. NPT4 is located at the apical side of renal tubules, and functions as an apical voltage-driven urate efflux transporter, also known as NPT4-Na^+^/phosphate co-transporter (Jutabha et al., 2010).

##### Function of NPT4 (*SLC17A3*)

NPT4 plays an important role in the urate excretion and operates functionally with basolateral organic anion transporters 1/3 (OAT1/OAT3) (Jutabha et al., 2010; Riches et al., 2009; Polasek et al., 2010; Jutabha et al., 2011). SUA is taken up by OAT1/OAT3 into tubular cell, then intracellular UA is excreted by NPT4 into the urinary lumen (Møller and Sheikh, 1982; Pritchard and Miller, 1993; Jutabha et al., 2010).

##### Mutation Loci of the NPT4 (*SLC17A3*) Gene

NPT4 variations have a greater effect on SUA concentration in women, have no close relationship with SUA in men (Jutabha et al., 2010). NPT4L and NPT4S are two splice variants of NPT4 (Polasek et al., 2010). NPT4L acts through the outlet channel of the proximal membrane of the renal proximal tubule. According to the voltage-driven promotion mechanism of NPT4L and its location on the proximal membrane of the proximal tubule, deeming NPT4 is the main channel for excretion of drugs and UA (Jutabha et al., 2010). Some reports have shown there were significant correlations between *rs9393672* and *rs942379* in NPT4 gene polymorphisms as well as changes in female SUA concentration (Jutabha et al., 2010; Riches et al., 2009).

#### OAT1 (*SLC22A6*) and OAT3 (*SLC22A8*)

Both OAT1 (*SLC22A6*) and OAT3 (*SLC22A8*), as urate/dicarboxylate exchangers, are located on the basolateral side of the proximal tubule (So and Thorens, 2010). Previous studies find that knockout of OAT1 or OAT3 slightly reduces uricosuria, indicating that their essential function is urate excretion (Eraly et al., 2008).

#### OAT2 (*SLC22A7*)

Members of the solute carrier 22A (*SLC22A*) family known as OATs, including OAT2, are expressed in various organs (Kimoto et al., 2018; Mathialagan et al., 2018). Among OATs, only OAT2 has a general expression pattern and is expressed in many tissues, such as the choroid plexus, liver, placenta, skeletal muscle, and kidney (Sager et al., 2018). In the kidney, OAT2 is located in the basolateral side of the proximal tubule for urate uptake transporter (Sakurai, 2013).

##### Function of OAT2 (*SLC22A7*)

OAT2 is a transporter of several known exogenous drugs and endogenous compounds (Kimoto et al., 2018; Mathialagan et al., 2018). Urate and cGMP may be substrates of OAT1, OAT2, and OAT3 (Henjakovic et al., 2015). And OAT2 could take up urate from blood to the proximal tubular cell. However, different from the other two transporters, OAT2 is relatively independent and not associated with the pH of the body. A recent study has shown that OATs, especially OAT2, contribute to creatinine transport (Sager et al., 2018). Although existing evidence shows that OAT2 is mainly expressed in the kidney, but it also plays a role in the liver (Vildhede et al., 2018).

##### Mutation Loci of the OAT2 (*SLC22A7*) Gene

Three quarters of OAT2 SNPs are found in men, and among the populations with these SNPs, the major allele frequencies of *C329T*, *G571A*, and *G1520A* were 0.94, 0.94, and 0.95, respectively (Xu et al., 2005). Three individuals (each with a non-synonymous SNP) are heterozygous at these loci, and originated from Sub-Saharan Africa (*C329T*), India-Pakistan (*G571A*), and Japan (*G1520A*) (Xu et al., 2005). OAT2 encodes a key renal solute transport protein, and genetic variations of *TBX2* are a determinant of CKD (Kato et al., 2015).

#### OAT4 (*SLC22A11*)

Organic anion determines the transport of most renal tubules and the secretion or reabsorption of substances (Hagos et al., 2007). Members of OAT family have a main task of handling drug complexes or exogenous secretions (Wright and Dantzler, 2004; Anzai et al., 2006). OATs are expressed along the proximal tubules of the kidney and other marginal epithelia, such as the blood-brain barrier, choroid plexus, and placenta (Ugele et al., 2003). OAT4 is identified as an apical transporter in proximal tubule cells and only expressed in advanced primates, including humans (Hagos et al., 2007).

##### Function of OAT4 (*SLC22A11*)

In addition to uric acid, OAT4 also promotes the absorption of high-affinity binding steroids such as estronesulfate (ES) or dehydroepian drosterone sulfate (Hagos et al., 2007). OAT4 can use chloride ion as the exchange anion of ES and uric acid (Roch-Ramel et al., 1994; Hagos et al., 2007). Physiologically, OAT4 guides the ion exchange of the proximal tubule through PAH/Cl^−^, PAH/ES, and possibly PAH/UA to excrete UA (Hagos et al., 2007). In previous studies, after the removal of sodium or addition of the NHE3-specific inhibitor amiloride, the ES uptake of HEK293-OAT4 cells is significantly reduced, indicating that OAT4 transport may be coupled with the effect of NHE3 (Lang et al., 2003; Tom Nijenhuis et al., 2005). OAT4 interacts with NHE3 and sodium dicarboxylate transporter 1 to participate in the maintenance of intracellular *α*-ketoglutarate (Hagos et al., 2007).

##### Mutation Loci of the OAT4 (*SLC22A11*) Gene

*SLC22A11 rs2078267* is associated with gout in some Europeans (van der Harst et al., 2010; Flynn et al., 2013; Köttgen et al., 2013). Previous studies show that *rs218657*1 is associated with SUA levels in the Pacific Micronesian population of Kosrae (Kenny et al., 2011; Flynn et al., 2013). *rs17299124* gene mutation is related to gout in Southeast Asians patients. Another study indicates that *rs17300741*, a common variant of OAT4/*SLC22A11*, is associated with the renal under excretion type gout (Sakiyama et al., 2014).

#### URAT1 (*SLC22A12*)

The *SLC22A12* gene encodes a transporter protein known as URAT1, which is a 553 amino acid protein that is 30% identical to rat organic cation transporter 1 at the amino acid level (Hosoyamada et al., 2004). URAT1 has been identified as a uric acid anion exchanger that affects UA homeostasis via urate reabsorption in human kidney (Enomoto et al., 2002; Zhou et al., 2010; Skwara et al., 2017; Misawa et al., 2020). It is expressed in the apical membrane of the proximal tubule of the human kidney (Hosoyamada et al., 2004).

##### Function of URAT1 (*SLC22A12*)

URAT1 plays an important role in urate reabsorption in the kidney. Sodium hydrogen exchange regulator (NHERF) protein, which is abundantly expressed in the apical membrane transported by epithelial cells, such as renal proximal tubules and small intestine, interacts with mURAT1 and plays an important role in the regulation of uric acid transport in renal proximal tubule cells (Cunningham et al., 2007). NHERF-1 deficiency directly affects uric acid absorption (Cunningham et al., 2007). Human URAT1 has the C-terminal sequence of T-Q-F, and cell analysis revealed that hURAT1 can specifically bind with NHERF-3 (Hosoyamada et al., 2004; Cunningham et al., 2007). NHERF-1 has been shown to play an important role in determining the cellular distribution of mURAT1 (Cunningham et al., 2007). A possible mechanism is that NHERF-1 may act as a partner in a manner similar to the adaptor proteins CAL and CFTR, as a membrane retention signal for stabilising mURAT1 in the plasma membrane, assuming the interaction between NHERF-1 and CFTRNpt2a, or as the determinant of mURAT1 circulation to the plasma membrane (Shenolikar et al., 2002).

##### Mutation Loci of the URAT1 (*SLC22A12*) Gene

Dysfunctional variants in URAT1 are considered as the major cause of hyperuricaemia (Zhu et al., 2021). *SLC22A12* produces a genetic variation that contributes to urate absorption and is a key factor in hyperuricaemia and gout (Toyoda et al., 2015; Tu et al., 2016). *SLC22A12 rs475688*(C/C) and p. N82N are reported to be significantly associated with gouty risk (Pavelcova et al., 2021). Interestingly, inactivating mutations in URAT1 have been shown to cause renal hypouricemia. Mutations in the *SLC22A12* gene can reduce SUA levels (Misawa et al., 2020). The results of this study shows that: 1) based on the serum levels of urate salt gene, the lack of *SLC22A12* was 10% more prominent in men than in women, and can be genetic (Misawa et al., 2020); 2) based on urate absorption, some of the variants truncate a protein that may have a termination codon that leads to the loss of URAT1 function (Cha et al., 2019).

### ABCG2

The ATP binding cassette subfamily member 2 (ABCG2), a multi-specific transporter, is located on the apical membrane in tissues including kidney and intestine. It exerts a critical physiological effect in the excretion of UA in the kidney and intestine (Maliepaard et al., 2001; Yun-Hong Lu et al., 2020).

#### Function of ABCG2

The export process of ABCG2 is ATP-dependent and unsaturated at the physiological concentration of UA, indicating that ABCG2 has high-capacity urate transport activity (Matsuo et al., 2009). ABCG2 dysfunction caused by common variants will significantly increase the risk of hyperuricaemia, and the reduction of extra renal urate excretion through dysfunctional ABCG2 is a common mechanism of hyperuricaemia (Ichida et al., 2012). One of the main causes of hyperuricaemia is not true overproduction of UA, but insufficient excretion of extra renal UA due to common ABCG2 dysfunction (Ichida et al., 2012). It exerts active roles in patients with CKD (Yano et al., 2014; Nigam, 2015; Bhatnagar et al., 2016). ABCG2 dysfunction enhances UA level and promotes renal dysfunction in CKD patients as well as systemic inflammatory responses (Bhatnagar et al., 2016; Cleophas et al., 2017).

In CKD, renal urate excretory mechanisms are compromised due to loss of renal function. In addition to transporting nucleotide analogues, ABCG2 is a vital transporter in intestinal UA excretion (Matsuo et al., 2009). Interestingly, renal urate excretion decrease but intestinal expression of ABCG2 increases, suggesting some sort of remote regulation of intestinal urate transport when renal transport is compromised. In CKD, intestinal ABCG2 becomes much more important, suggesting remote organ communication between the injured kidney and the intestine (Nigam and Bhatnagar, 2018).

#### Mutation Loci of the ABCG2 Gene

To investigate ABCG2 gene mutations in hyperuricaemia patients, researching performed mutation analysis on all coding regions and intronexon boundaries of the ABCG2 gene in 90 Japanese patients with hyperuricaemia (Matsuo et al., 2009). The following six asynchronous mutations were found: *V12M*, *Q126X*, *Q141K*, *G268R*, *S441N*, and *F506SfsX4* (Matsuo et al., 2009). Among them, the allele frequency of *Q141K*, *V12M*, and *Q126X* is 31.9, 19.2, and 2.8% (Matsuo et al., 2009). ABCG2 gene mutation has a significant effect on UA, especially the amino acid substitution in *Q141K*, which leads to risk of hyperuricaemia and gout (Wen et al., 2015; Cleophas et al., 2017). At the same time, decrease in the amount of ABCG2 protein will inhibit *Q141K* activity (Kondo et al., 2004). In addition, ABCG2 *rs2231142* considered a risk allele for gout (Butler et al., 2021).

### PDZK1

Polyvalent PDZ domain 1 (PDZK1) is a multi-domain protein containing four PDZ domain tubular cells observed in the apical membrane of the kidney proximal tubule. It is highly expressed at the apical membrane of tubular epithelial cells, and that most of the above-mentioned apical transporters have been reported to directly interact with PDZK1 (Prestin et al., 2017).

#### Function of PDZK1

PDZK1 acts as a scaffold protein to regulate the activity of various transport proteins including URAT1 and NPT1 in the proximal tubules (Miyazaki et al., 2005; Lu et al., 2019). Using a yeast two-hybrid screen system, found that PDZK1 regulates the functional activity of URAT1 and enhances its UA reabsorption capacity (Anzai et al., 2004). Furthermore, PDZK1 might be an important upstream molecule of ABCG2, which changes its function in the small intestine (Lu et al., 2019). Soluble UA induced upregulation of ABCG2 expression and function in intestinal cell lines is dependent on PDZK1 at the transcriptional level (Lu et al., 2019). However, the correlation between PDZK1 and ABCG2 needs to be further investigated. SMCT1 (*SLC5A8*), a high-affinity lactate transport system that interacts with PDZK1, plays an important role in the reabsorption of urate in human kidney (Gopal et al., 2004; Miyauchi et al., 2004; Otsuka et al., 2019). *rs12129861* in PDZK1 is considered a risk allele for gout (Butler et al., 2021). According to previous studies, PDZK1 affects urate transporters and thus may have a certain effect on various transporters, but it is not yet possible to determine its specific mode of action.

## Urate-Lowering Drugs in Hyperuricaemia-Related Diseases

At present, there have been many studies on the traditional treatment of hyperuricaemia (Yang et al., 2018). The discovery of urates transporters provides new ideas for the development of drugs for the research of hyperuricaemia. In addition to urate transport inhibitors, xanthine oxidase inhibitors, as the most popular drug candidates, have attracted a lot of attention (Figure 2). Xanthine oxidoreductase is a rate-limiting enzyme catalyzing formation of UA by oxidative hydroxylation of hypoxanthine and xanthine in purine metabolism (Battelli et al., 2014). It plays a vital role in the production of hyperuricaemia and gout (Nakatani et al., 2021). We summarize advances in research on urate -lowering drugs including urate transporter inhibitors, xanthine oxidase inhibitors as well as novel urate -lowering drugs, and evaluate the effect of urate-lowering therapy on the rate of hyperuricaemia-related diseases.

### Urate Transporter Inhibitors

#### URAT1 Inhibitors

##### Arhalofenate

Arhalofenate, an emerging URAT1 inhibitor, is a dual-acting agent (Shahid and Singh, 2015). In addition to increasing uric acid excretion, it inhibits the production of IL-1β, thereby reducing the occurrence of flare gout (Dalbeth et al., 2014). Arhalofenate increases urate excretion by inhibiting the action of URAT1, which is one of its main effects (Shahid and Singh, 2015). The other main roles of arhalofenate are to activate AMPK, inhibit NF-κB and NLRP3 inflammasomes to promote the polarisation of anti-inflammatory macrophages, as well as significantly reduce UA-induced inflammation (Salminen et al., 2011; Wang et al., 2016). There are similar studies showing that arhalofenate acid activates AMPK in macrophages *in vitro*, inhibits the activation of NLRP3 inflammasomes, and reduces MSU-induced IL-1β production (Kim et al., 2016; McWherter et al., 2018). Moreover, arhalofenate acid activates AMPK downstream targets to participate in the regulation of mitochondrial function and maintain the function of mitochondrial crest (McWherter et al., 2018).

Colchicine can inhibit the release of IL-1β-induced MSU crystal deposition joints. Therefore, researchers believe that in the next few years, arhalofenate may become a substitute for colchicine (Shahid and Singh, 2015). In addition, arhalofenate lowers blood lipid and blood sugar levels in patients with hypertriglyceridaemia and diabetes. Owing to its effect on OAT4, it can reduce uric acid level in hypertensive patients treated with diuretics, and diuretics, especially thiazide drugs, and prevent hyperuricaemia (Shahid and Singh, 2015).

##### Lesinurad

Lesinurad (RDEA594) is a selective inhibitor of URAT1 in the proximal tubules of the kidney (Shahid and Singh, 2015). RDEA594 through affects OAT4 relieve hyperuricaemia which caused by diuretics; however, lesinurad has no effect on other transport molecules, such as OAT1 and OAT3 (Shahid and Singh, 2015). RDEA594 in combination with XOI is a new treatment option considered for gout (Engel et al., 2017). Verinurad (RDEA3170), a new URAT1 inhibitor, demonstrates high potency in inhibiting URAT1 (Martin et al., 2018; Yue et al., 2019). *In vitro* studies have shown that three times the potency of benzbromarone and 100 times of probenecid effectiveness (Yue et al., 2019). RDEA3170 is currently in phase II clinical trials for the treatment of gout and asymptomatic hyperuricaemia (Yue et al., 2019).

##### Losartan

Losartan, an angiotensin II receptor blocker (ARB), is confirmed to reduce SUA level (Matsumura et al., 2015; Bryant et al., 2021). It is due to its inhibition of urate/anion exchanger on the brush border membrane of renal proximal tubular epithelial cells (Enomoto et al., 2002; Iwanaga et al., 2007). The urate transporter URAT1 participates in the reabsorption of UA from lumen to cytoplasm along proximal tubules. Losartan lows the urate reabsorption by inhibitory activity of URAT1 in the range of clinically relevant concentrations (0.1–10 nm) (Iwanaga et al., 2007). These results suggest that losartan is effective inhibitors of URAT1, which may explain why patients taking losartan generally have low UA levels (Iwanaga et al., 2007). Intriguingly, UA level will restore at higher concentrations of losartan mainly due to trans-stimulation of these ARBs at higher concentrations (Iwanaga et al., 2007). Nevertheless, one recent study finds that the urate reduction is not a class effect of ARBs. Compared with multiple ARBs in cluding candesartan, valsartan, azilsartan, eprosartan andirbesartan, only losartan has clear evidence of its ability to lower SUA level. This result suggests that for patients with hypertension and hyperuricaemia, losartan could be regarded as a first-line agent with irbesartan as an alternative when appropriate (Sutton Burke et al., 2020).

### Anti-inflammatory Drug

#### Nonsteroidal Anti-inflammatory Drug (NSAIDs)

NSAIDs including aspirin and steroids are widely used for pain relief and inflammatory suppression during acute gout attacks (Enomoto et al., 2002; Ragab et al., 2017). Accumulating studies indicate that affect renal urate excretion in an inverse dose dependent manner. High dose aspirin is uricosuric, while low dose causes urate retention (Roch-Ramel et al., 1997; Enomoto et al., 2002). According to previous reports, the mechanism underlying dual effects of aspirin involve the renal urate transporter URAT1 (Choi et al., 2005; Zhang et al., 2014). High dose aspirin decreases SUA level by inhibiting URAT1, whereas low dose exerts urate retentive role through stimulating URAT1 (Zhang et al., 2014). Low dose of salicylate (75, 150, and 325 mg per day) reduces urinary urate excretion and contributes to gouty risk (Caspi et al., 2000). However, there was no significant change in SUA levels and urinary urate excretion in patients with gout who received 325 mg of aspirin daily combined with probenecid. Therefore, urate-lowering drugs (e.g., uricosuric agents orxanthine oxidase inhibitors) may reduce the effect of low-dose aspirin on hyperuricaemia (Harris et al., 2000; Zhang et al., 2014).

##### Glucocorticoids

Glucocorticoids, such as dexamethasone (DEX), have been shown to increase xanthine oxidase activity in rats (Patel et al., 2014). Besides, DEX significantly increases the renal excretion of urate (Li et al., 2019). Many membrane transporters are involved in the urate reabsorption and secretion (Hyndman et al., 2016). During secretion by the renal tubules, SUA is absorbed by OAT1 and OAT3 to enter the renal tubule cells, and then enters urine through NPT1, NPT4, MRP4, BCRP, as well as other efflux transporters. Although DEX increases NPT1 and NPT4, it significantly reduces OAT3 expression in mouse kidney, which also shows the complex roles of DEX on urate absorption (Li et al., 2019). Nonetheless, compared to urate reabsorption, the tubular secretion is only a minor component of urine UA excretion. OAT10, GLUT9, and URAT1 are apically absorbed transporters of urate and play a crucial role in the urate reabsorption (Li et al., 2019). DEX had no effect on OAT10 and GLUT9, but significantly reduced the mRNA level of URAT1. Therefore, DEX-mediated increase in uric acid excretion is mainly due to the downregulation effect of URAT1 (Li et al., 2019).

### SGLT2 Inhibitors

The urine UA and SUA lowering effects of SGLT2 inhibitors are similar (Vallon and Thomson, 2017; Nespoux and Vallon, 2020). Researchers use SGLT2-, SGLT1-, URAT1-, and GLUT9-knockout mouse models to investigate the UA-lowering effect of the SGLT2 inhibitor canagliflozin, and show that the mechanism of this effect involved intraluminal glucose transmission and inhibition of the proximal tubule urate transporter URAT1 (Novikov et al., 2019; Novikov et al., 2019). Insulin enhances the activity of URAT1. Therefore, the inhibition of SGLT2 may reduce the activity of URAT1 by enhancing luminal glucose transmission or lowering insulin levels, or SGLT2 and URAT1 may functionally interact in the proximal tubule, thereby inhibiting SGLT2 to partially suppress URAT1 (Novikov et al., 2019; Nespoux and Vallon, 2020). These SGLT2 inhibitors may also help protect mitochondrial function and tubular cell metabolism (Nespoux and Vallon, 2020). Preliminary studies in patients with T1DM and T2DM have shown that diabetes increases the urine ratio of lactobionate, and this may reflect the metabolic process of mitochondrial oxidation to glycolysis, which is reversed by SGLT2 inhibitors (Nespoux and Vallon, 2020).

### ABCG2 Inhibitors

Febuxostat is a common drug for the treatment of gout. Its main function is to inhibit xanthine oxidase. One study indicates that febuxostat exerts strong inhibitory effect on ABCG2 (Miyata et al., 2016). The researchers find that use of febuxostat reduces the efflux of many drugs mediated by ABCG2 and prolongs the action time of the drug in the body without increasing the level of UA. Further analysis show that the inhibitory effect of febuxostat on ABCG2 occurs both at clinical concentration *in vitro* and in mouse intestinal tract. It was also demonstrated that febuxostat can enhance the intestinal absorption of a substrate of ABCG2 (Yamasaki et al., 2008; Miyata et al., 2016). It is worth noting that febuxostat inhibits ABCG2 more strongly than the two known ABCG2 inhibitors (Ko143 and elacrida), which means that febuxostat has not only advantages over these two inhibitors but also stronger in safety and ability than other ABCG2 inhibitor (Allen et al., 2002). However, decreased ABCG2 function may enhance the risk of hyperuricaemia, genetically. Thus, these findings suggest novel potential applications and risks in clinical use of febuxostat.

### Xanthine Oxidase Inhibitors

#### Allopurinol

Allopurinol, a xanthine oxidase inhibitor, is one of first-line drugs for the management of gout (Stamp et al., 2016; Day et al., 2017; Coombs et al., 2021). Allopurinol has been used widely for many years, however, the reduction and maintenance of blood urate concentrations is often not achieved (Day et al., 2017). The main cause may be linked to intersubject variation in allopurinol pharmacokinetics and pharmacodynamics (Wright et al., 2013; Wright et al., 2016). In addition, recent studies report that high-dose allopurinol may induce severe cutaneous adverse drug reactions and liver injury. High drug-dosage also lead to high mortality in patients with CKD (Huang et al., 2021). Available findings suggest that the initial dosage of allopurinol should be low, particularly in patients with renal impairment. The dose should then be increased slowly until sufficient to dissolve MUS (Day et al., 2017).

#### EEAK

The xanthine oxidase inhibitor tetrapeptide EEAK is identified from the skeletal myosin of tuna (Yu et al., 2021). Inhibitory peptides from tuna protein simulations indicate that traditional hydrogen bond interactions, attractive charge interactions, and salt bridges play an important role in the interaction of EEAK with key residues of xanthine oxidase (eg, Glu802, Arg880, and Glu1261) (Yu et al., 2021). Overall, the current work strongly suggests that the tetrapeptide EEAK may be a promising compound as a natural inhibitor of xanthine oxidase for the control of gout and hyperuricaemia (Yu et al., 2021).

#### Rhodiola Crenulata Extracts and Their Phytochemicals

Rhodiola crenulata is an important member of the genus Rhodiola, mainly distributed in northwest China. Previous studies have proved that the root of Rhodiola crenulata has beneficial properties, including scavenging active oxygen substances and anti-Alheimer’s disease (Chen et al., 2012; Zhang et al., 2013). Researchers use fractional distillation techniques to separate four phytochemicals from Rhodiolarosea extract. These compounds are identified as 4′-hydroxyacetophenone (4-HAP), epicatechin-(4β,8)-epicatechingallate (B2-3′-O-gallate), red Sedum and *p*-tyrosol used mass spectrometry and nuclear magnetic resonance spectroscopy (Chu et al., 2014). These purified compounds are then evaluated for their inhibitory effects on xanthine oxidase activity and compared with known XO inhibitors (allopurinol). The results show that 4-HAP and B2-3′-O-gallate are effective xanthine oxidase inhibitors (Chu et al., 2014).

#### 2-[4-Alkoxy-3-(1H-tetrazol-1-yl)phenyl]-6-oxo-1,6-dihydropyrimidine-5-carboxylic Acid Derivatives

The researchers in this study designed and synthesized a series of 2-[4-alkoxy-3-(1H-tetrazol-1-yl)phenyl]-6-oxo-1,6-dihydropyrimidine-5-carboxylic acid derivatives (8a-8z) and further evaluated their inhibitory effect on xanthine oxidase *in vitro* (Zhang et al., 2019). The results show that all test compounds (8a-8z) showed significant xanthine oxidase inhibitory efficacy (Zhang et al., 2019). Among them, compound 8u becomes the most effective xanthine oxidase inhibitor, with an IC50 value of 0.0288 mM, which is equivalent to febuxostat (IC50 ¼ 0.0236 mM) (Zhang et al., 2019). In addition, acute oral toxicity experiments in mice showed that compound 8u is non-toxic and can tolerate doses up to 2000 mg/kg (Zhang et al., 2019). Therefore, compound 8u may be a potentially effective xanthine oxidase inhibitor for the treatment of hyperuricaemia with low toxicity (Zhang et al., 2019).

## Other Drugs That Affect UA Levels

### Favipiravir

Favipiravir is an antiviral agent that inhibits the RNA-dependent RNA polymerase of many RNA viruses (Furuta et al., 2017). Favipiravir is metabolized by aldehyde oxidase and xanthine oxidase to the inactive metabolite M1, which is excreted into urine. In the kidney, the processing of uric acid is regulated by the balance between proximal tubule reabsorption and renal tubule secretion. Favipiravir and M1 are moderate inhibitors of OAT1 and OAT3, which are involved in the excretion of uric acid in the kidney (Mishima et al., 2020). In addition, M1 enhances uric acid reabsorption through URAT1 in the proximal tubule of the kidney (Mishima et al., 2020). Therefore, it is believed that favipiravir reduces the excretion of urate in the urine, leading to increase the level of SUA (Mishima et al., 2020).

### PF-06743649

Some drugs under development have shown dual inhibitory effects on XOD and URAT1. But little information is available (Yue et al., 2019). Among of them, PF-06743649 is the first drug to enter clinical trials with dual effects on XOD and URAT1. PF-06743649 phase I clinical trials have been completed (Yue et al., 2019). Clinical studies have shown that PF-06743649 causes a large and rapid decrease in serum uric acid in healthy subjects and gout patients (Yue et al., 2019).

## Conclusion

NPT1, NPT4, ABCG2 expressed on the apical membrane and OAT1, OAT3 expressed on the basolateral membrane have been confirmed to contribute to the secretory transport of urate from proximal tubular epithelial cells into the tubule lumen (Cleophas et al., 2017). URAT1, GLUT9, OAT4 localized on the apical membrane are responsible for UA reabsorption from the tubule lumen to proximal tubule epithelial cells (Auberson et al., 2018; Wen et al., 2020). NPT1, URAT1, and OAT4 are known to bind to PDZK1 through their C-terminal PDZ domain. The metabolic disorder of UA mainly linked with abnormal urate transporters is an important cause of many diseases. In recent years, an increasing number of studies have shown that elucidating the urate transporters is essential to address the balance of urate homeostasis and hyperuricaemia-related diseases. In this review, eleven transporters and urate lowering drugs are summarized and evaluated.

Generally, asymptomatic hyperuricaemia is not an indication for treatment to lower the SUA level in persons with normal renal function. The recommended first line of urate‐lowering therapy includes the xanthine oxidase inhibitors allopurinol and febuxostat by reducing urate. The novel uricosurics agent including lesinurad, arhalofenate, canagliflozin, xanthine oxidase inhibitor tetrapeptide EEAK, rhodiola crenulata, favipiravir, PF-06743649 increase renal urate excretion by inhibiting reabsorption. In sum, the exploration of the urate transports and inhibitors can enhance our understanding of hyperuricaemia and hyperuricaemia-related diseases. It may provide essential references or cross references to be contributed to further elucidate urate-lowering drugs benefits as well as provide theoretical support for the scientific research on hyperuricemia and related diseases.

## Data Availability

The raw data supporting the conclusions of this article will be made available by the authors, without undue reservation.
